# Three-stage fermentation of the feed and the application on weaned piglets

**DOI:** 10.3389/fvets.2023.1123563

**Published:** 2023-02-16

**Authors:** Dahai Jiang, Manqi Yang, Jun Xu, Liping Deng, Cong Hu, Liangliang Zhang, Yunzhang Sun, Jianchun Jiang, Liming Lu

**Affiliations:** ^1^Academy of Advanced Carbon Conversion Technology, Huaqiao University, Xiamen, China; ^2^College of Chemical Engineering, Huaqiao University, Xiamen, China; ^3^Zhangzhou DaBeiNong Agriculture and Husbandry Science & Technology Co., Ltd., Zhangzhou, China; ^4^Jiangxi DaBeiNong Technology Co., Ltd., Nanchang, China; ^5^Beijing DaBeiNong Technology Group Co., Ltd., Beijing, China; ^6^The Key Laboratory of Healthy Mariculture for the East China Sea, Ministry of Agriculture, Fisheries College, Jimei University, Xiamen, China; ^7^Institute of Chemical Industry of Forest Products, CAF, Nanjing, China

**Keywords:** probiotics, nutritional value, weaned piglets, growth performance, fermented feed

## Abstract

Numerous studies have demonstrated that soybean meal (SBM) contains high levels of anti-nutritional factors, which interrupt gastrointestinal homeostasis or metabolism normally of the weaned piglets. Here, the mixed probiotics, including *Bacillus licheniformis* (*B. licheniformis*, CGMCC 8147), *Saccharomyces cerevisiae* H11 (*S. cerevisiae* H11) and *Lactobacillus casei* (*L. casei*, CGMCC 8149) were applied to the three-stage fermentation of functional feed. Our research investigated the optimum ratio of inoculation, optimal time of inoculation, combination of substrates, and nutritional value of the fermented feed. The optimal microbial combination was *B*. *licheniformis*: *S*. *cerevisiae*: *L*. *casei* = 2:2:1, inoculating at 0, 12 and 24 h, respectively. The results revealed that crude protein and acid-soluble protein were remarkably improved and had lower pH. Trypsin inhibitor, glycinin and β-conglycinin were reduced by 79.86, 77.18, and 69.29%, respectively. Moreover, animal trials further evaluated the growth-promoting effects of the fermented feed. It was noted that the average daily gain of weaned piglets was significantly higher, and the ratio of feed with weight, diarrhea incidence and mortality were lower significantly. The concentrations of serum immunoglobulin G(IgG), IgA, IgM, Complement C3 and interferon-γ (IFN-γ), and lysozyme activity were all increased. The relative abundance of fecal microbiota improved, especially *lactobacillus*, which increased the abundance of fecal dominant probiotics. Overall, the fermented feed may be conducive to the growth and health of weaned piglets by improving nutritional value, immunity properties, relative abundance of fecal microflora, and decreasing anti-nutritional factors of feed, thereby making them viable and usable feedstuffs for potential use in livestock industries.

## 1. Introduction

In the swine industry, feed is the most important cost source, which accounts for 60–70 percent. The main components of the current feed are corn and soybean meal (SBM) which are the main sources of nitrogen. But, due to some anti-nutritional factors including antigenic proteins in the SBM, nutrients are not fully absorbed and utilized by piglets. Among them, glycinin and β-conglycinin are two major antigenic proteins that account for ~75% of the total soybean proteins and are responsible for allergic reactions (1, 2). Improving the comprehensive utilization of feed and decreasing the diarrhea incidence of weaned piglets have been an important task in commercial pig breeding.

Antibiotics can partially solve the problem of livestock and poultry diseases, and the proper application of antibiotics can effectively improve the survival rate of animals and increase economic benefits. The bacterial resistance and antibiotic residues caused by the overuse of antibiotics have become a serious threat to human health. Therefore, it is imperative to find other safe and effective substances to replace antibiotics. In July 2020, the Ministry of Agriculture and Rural Affairs of the People's Republic of China banned the addition of antibiotics to feed, and probiotics have been widely used as one of the alternatives. Many studies also showed that antigenic proteins in feed can be reduced and their bioavailability improved by means of microbial fermentation (3). Probiotics have been widely used as feed additives to promote the growth performance of animals (4). At present, the probiotics commonly used in fermented feed are mainly *Lactobacillus, Bacillus, Saccharomyces*, and *Moulds*. The positive effects of these probiotics on metabolism were associated with changes in the intestinal microbiota. In general, in order to have a better fermentation effect, two or more probiotics are mixed in a certain ratio, and the effect of feed fermentation is more significant through a synergistic effect. Multi-probiotic fermentation is the focus of research in the field of fermented feed in recent years and has been attracted by researchers.

Based on previous studies, our team screened and optimized strains of highly bioactive functional microorganisms, *Bacillus licheniformis* (*B. licheniformis*, CGMCC No. 8147), *Saccharomyces cerevisiae* H11(*S. cerevisiae*, preserved by laboratory) and *Lactobacillus casei* (*L. casei*, CGMCC No. 8149). This study aimed to explore an excellent fermented feed and evaluate the effects of fermented feed on growth performance, immunity properties and fecal microbiota of weaned piglets.

## 2. Methods

### 2.1. Bacterial strains

*B. licheniformis* and *L. casei* were isolated from the gut of a healthy pig, which have good stress resistance or inhibit pathogenic bacteria and other probiotic functions. *S. cerevisiae* was preserved by laboratory. *B. licheniformis, S. cerevisiae* and *L. casei* are government-authorized probiotics in China.

### 2.2. Design and optimization of the fermentation conditions

#### 2.2.1. Ratio of inoculation

The inoculation ratio is the main parameter of fermented feed for mixed strain combinations. Six sets of experiments with different ratios (*B. licheniformis, S. cerevisiae*, and *L. casei* at a ratio of 1: 1: 1, 1: 1: 2, 1: 2: 1, 1: 2: 2, 2: 1: 1, 2: 1: 2, 2: 2: 1) were designed with the content of glycinin and β-conglycinin as indicators to finally determine the optimal ratio of fermentation strains. The general feed without the strain was used as control.

#### 2.2.2. Time of inoculation

The oxygen conditions required by each strain are not consistent. Since *B*. *licheniformis* is aerobic, *S*. *cerevisiae* is partly anaerobic and *L*. *casei* is anaerobic, in order to give full play to the functions of various probiotics, they are added into the feed for fermentation according to the order of *B. licheniformis, S. cerevisiae* and *L. casei*, respectively at different times (0: 0: 0, 0: 0: 12, 0: 12: 12, 0: 12: 24 /h). The optimal time of inoculation was determined with the degradation of glycinin and β-conglycinin as indicators.

#### 2.2.3. Ratio of fermentation substrates

In this study, general feed such as corn, bran and SBM were used as the main fermentation substrates. In order to study the fermentation effect of the composite strains under different ratios of fermentation substrates, three sets of experiments with different ratios (corn: bran: SBM=1: 7: 2, 2: 6: 2, 3: 5: 2) were designed. The optimal ratio of fermentation substrates is determined according to the crude protein levels and the number of viable cells.

### 2.3. Production of fermented feed

**Figure 1:**
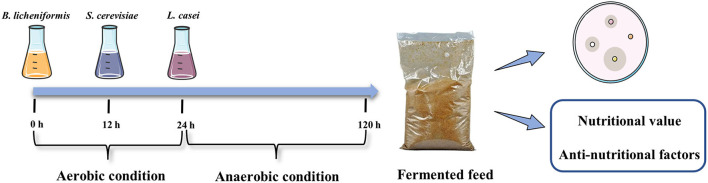
Production of fermented feed.

*B. licheniformis* was incubated in LB medium at 37°C for 48 h with 200 rounds per minute (rpm). *S. cerevisiae* was incubated in YPD medium at 30°C for 48 h with 200 rpm. *L. casei* was incubated in MRS medium at 37°C for 48 h with 200 rpm. The fermentation substrates were mixed at an optimal ratio. Then we mixed the mixtures with sterile water in a ratio of 25: 1 and controlled the water temperature at 33 ± 1°C. The wet mixed substrates were inoculated with well-activated *B. licheniformis, S. cerevisiae* and *L. casei*, 10% inoculum size, at different times, respectively. The fermented mixture was transferred to a fermentation bag and fermented at 33 ± 1°C for 96 h. The specific procedure is shown in Figure 1.

### 2.4. Analysis of chemistry

The samples of uninoculated and inoculated feed were analyzed. The content of trypsin inhibitor, glycinin and β-conglycinin in feed was analyzed using an ELISA kit (Longzhoufangke Bio Co., Ltd., Beijing, China). The content of crude protein and acid-soluble protein was in accordance with “GB/T 6432-2018” and “GB/T 22492-2008” from the national standard of the People's Republic of China, respectively. The pH values were measured using a portable HI 99163 pH meter (Hanna instruments, Woonsocket, RI, USA). Total acid and acetic acid were measured according to “GB/T 12456-2021”. The lactic acid content was determined using a lactic acid enzymology assay kit (Nanjing Jiancheng Bioengineering Institute Co., Ltd., Nanjing, China). For all counts, the conventional method was used by serially diluting samples with sterile water and plating out several dilutions onto culture plates, and an average of 3 plates was used for each tested dilution. At enumeration, the colonies counted on the plates ranged between 20 and 300 (5). The results were expressed as colony-forming units per gram (CFU/g). All the measurements of chemical analysis were performed in sextuplicate.

### 2.5. Animal treatment

**Figure 2:**
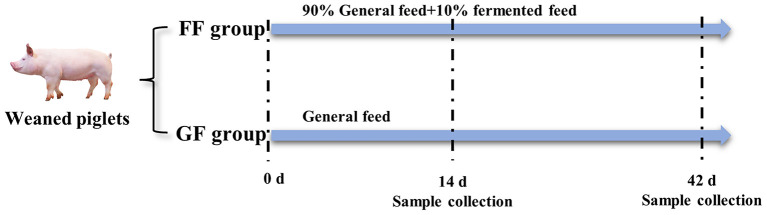
Treatment of weaned piglets (*n* = 120). The experiment was finished at the age of 42 days old.

All animal works were conducted according to the guidelines for the care and use of experimental animals established by the National Institute of Animal Health. The experimental animals were weaned piglets from Fujian Xingyuan Agricultural and Husbandry Co., Ltd. The commercial basal diet of feed was purchased from Zhangzhou DaBeiNong Agricultural and Husbandry Technology Co., Ltd., which mainly includes corn, bran and SBM. Experimental piglets were weaned at the age of 28 days. The house, feed trough and drinker were thoroughly cleaned and disinfected before starting the experiment. As shown in Figure 2, 120 weaned piglets (half males and half females) were divided into two groups (experimental and control group) with 60 replications for each group, totally random, and every group was allotted three treatments in a completely randomized design with 20 repetitions per treatment. The control group was fed by general feed (GF) and the experimental group was fed by fermented feed (FF) with a 10% addition. All the piglets were provided *ad libitum* access to water and feed according to Chinese biosecurity guidelines. The weaned piglets were continually fed diets for 42 days after weaning.

### 2.6. Analysis of the growth performance

The weaned piglets were weighed at the initial and terminal experiments after they fasted for 12 h. BW and feed intake were measured to calculate the average daily gain (ADG), average daily feed intake (ADFI), and the ratio of feed with weight (F: G). Diarrhea symptoms and mortality, if any, were recorded twice a day for each tagged piglet during rearing.

### 2.7. Analysis of immunological properties

Sterile disposable gloves were used during sample collection to avoid human contamination. At 05:00 on day 14 and 42, blood samples were collected in heparinized vacutainer tubes through the jugular vein from weaned piglets after fasting for 12 h. Two samples (1 male and 1 female) were taken from each treatment randomly, respectively, with a total of six samples from each group. The blood samples were centrifuged at 2,500 × *g* for 15 min at 4°C to obtain the serum samples and were kept at −80°C until analysis. The concentrations of total IgG, IgA, IgM, Complement C3 and interferon-γ (IFN-γ), and lysozyme activity in the plasma were measured using the pig ELISA quantification kit (Nanjing Jiancheng Bioengineering Institute Co., Ltd., Nanjing, China).

### 2.8. Estimation of fecal microbiota

For microbiological analyses, 50 g of fresh feces (on day 14 and 42) from weaned piglets were taken and immediately stored in sterile centrifuge tubes, from the experimental and control group respectively. Two samples (1 male and 1 female) were taken from each treatment randomly, respectively, with a total of six samples from each group. All fresh fecal samples were immediately stored in liquid nitrogen for transportation back to the laboratory and kept at −20°C until analysis using high-throughput sequencing technology. Briefly, total DNA of samples was extracted using a DNA extraction kit, and then, the V3+V4 regions of the 16S rRNA gene of bacteria was amplified and high-throughput sequenced on an Illumina HiSeq platform (Beijing Biomarker Biotechnology Co. Ltd, Beijing, China). The Sequencing data have been uploaded to GenBank (Accession number, PRJNA921733). Analysis of microbiota abundance and diversity was performed using BMKCloud (www.biocloud.net).

The QIIME 2 was used to calculate the community richness (Chao1 and Ace index) and community diversity (Shannon and Simpson index). These indicators were then visualized by the software platform and represented the community richness, evenness and diversity of fecal microbiota. To analyze Beta diversity, we used principal coordinates analysis (PCoA), non-metric multidimensional scaling (NMDS) and analysis of box plot (binary Jaccard distance) to show the effect of FF on piglet fecal microbiota.

### 2.9. Statistical analysis

All data are presented as the mean ± standard deviation (SD) and compared by one-way ANOVA and *t*-test using the SPSS 25.0 software (Chicago, USA). A *p*-value <0.05 was considered significant, and a *p*-value <0.01 was considered extremely significant.

## 3. Results

### 3.1. Optimization of fermentation conditions

As shown in Table 1, the best microbial proportion of *B*. *licheniformis, S*. *cerevisiae* and *L*. *casei* was 2: 2: 1 in group 8, in which the lowest content of glycinin and β-conglycinin was observed. Compared with the control (group 1) without the added probiotics, glycinin and β-conglycinin were decreased by 41.0 and 31.6%, respectively.

**Table 1:** Effect of the rate of inoculation on glycinin and β-conglycinin contents (*n* = 6).

**Number**	**Ratio**	**glycinin**	**β-conglycinin**
1	0	146.39 ± 3.05^a^	121.01 ± 1.57^a^, ^b^
2	1: 1: 1	106.91 ± 1.27^b^	101.56 ± 0.77^b^
3	1: 1: 2	91.86 ± 0.09^d^	86.74 ± 1.91^a^, ^b^
4	1: 2: 1	91.75 ± 0.72^d^	90.24 ± 1.43^b^, ^c^
5	1: 2: 2	94.51 ± 1.05^d^	87.96 ± 0.93^a^
6	2: 1: 1	100.82 ± 0.65^c^	82.23 ± 0.39^c^
7	2: 1: 2	89.27 ± 0.32^e^	85.39 ± 0.73^c^
8	2: 2: 1	86.30 ± 0.68^e^	82.75 ± 3.96^c^

a − e With a column, the letter means without a common superscript difference (*p* < 0.05).

To further determine the optimal inoculation order, we inoculated *B. licheniformis, S*. *cerevisiae* and *L. casei* at different times respectively. Compared with the control group, the degradation rates of both glycinin (59.5%) and β-conglycinin (79.5%) were more favorably increased, especially in group 5 (Table 2).

**Table 2:** Effect of timing of inoculation on glycinin and β-conglycinin contents (*n* = 6).

**Number**	**Time**	**glycinin**	**β-conglycinin**
1	Control	146.39 ± 3.05^a^	121.01 ± 1.57^a^
2	0: 0: 0	97.76 ± 0.67^b^	87.22 ± 2.75^c^
3	0: 0: 12	95.93 ± 0.44^b^	94.96 ± 2.49^b^
4	0: 12: 12	61.86 ± 5.03^c^	26.12 ± 0.36^d^
5	0: 12: 24	59.22 ± 1.19^c^	24.83 ± 1.39^d^

a − d Within a column, the letter means without a common superscript difference (*p* < 0.05).

According to the experimental result (Table 3), we found that the ratio of crude protein improvement and the CFU in group 2 and group 3 were higher than those in group 1. However, the difference between the results of group 2 and group 3 was very small. We used the scheme of group 2 because of its economy and practicality in the following study.

**Table 3:** Effect of fermentation substrates (*n* = 6).

**Item**	**Group 1**	**Group 2**	**Group 3**
Corn: Bran: SBM	1: 7: 2	2: 6: 2	3: 5: 2
Rate of crude protein enhancement/%	5.58 ± 0.12^b^	7.92 ± 0.03^a^	8.03 ± 0.03^a^
10^6^ CFU/g	6.5 ± 0.16^b^	6.98 ± 0.05^a^	7.05 ± 0.09^a^

a, b Within a row, the letter means without a common superscript difference (*p* < 0.05); No letter means the difference is not significant (*p* > 0.05).

### 3.2. Nutritional value and anti-nutritional factors of the fermented feed

**Figure 3:**
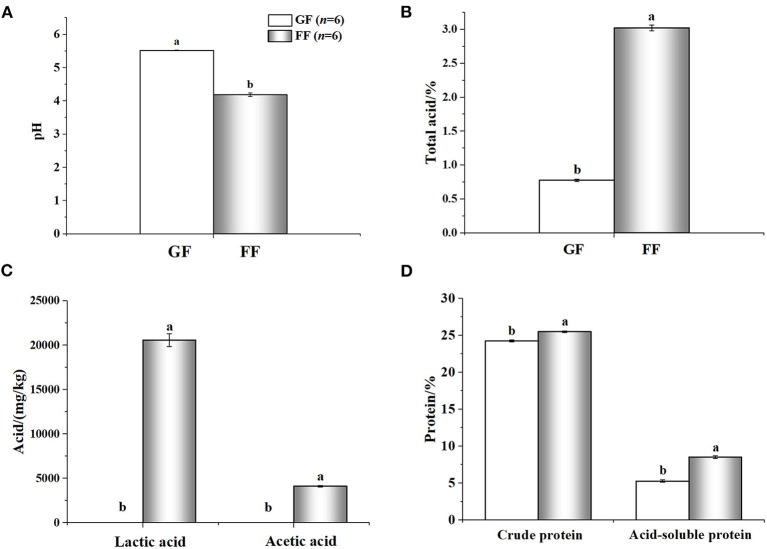
Analyzed nutrient composition [**(A)**: pH; **(B)**: total acid; **(C)**: acid; **(D)**: protein] of fermented feed and general feed. ^a,b^ Within the same parameter, superscripts with different letters indicate a significant difference (*p* < 0.05); The rest are as above.

**Figure 4:**
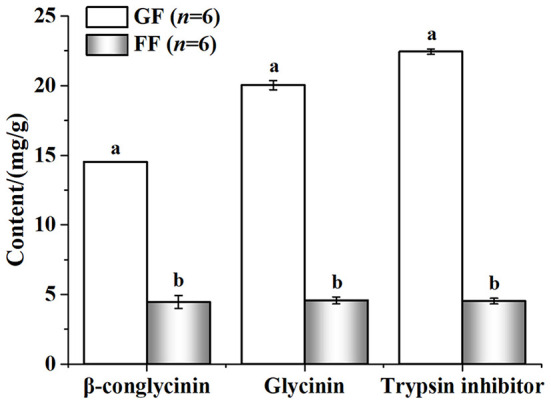
Effect of fermentation on the concentration of anti-nutritional factors.

The nutritional value of the FF was shown in Figure 3. Compared with GF, FF showed lower pH that total acid significantly increased. Large amounts of lactic (20590.01 ± 721.60707 mg/kg) and acetic acid (4116.24 ± 85.40485 mg/kg) were also detected in FF, while none were found in GF. The content of crude protein and acid-soluble protein also improved, significantly. Additionally, viable cells of *B*. *licheniformis, S*. *cerevisiae* and *L*. *casei* reached 2.8 × 10^6^, 2.3 × 10^6^ and 4.3 × 10^6^ CFU/g in FF, respectively (data not shown). As shown in Figure 4, trypsin inhibitor, glycinin and β-conglycinin can be found a significant decrease in FF compared with GF, reduced by 79.86, 77.18, and 69.29%, respectively.

### 3.3. Growth performance

As shown in Table 4, in general, the growth performance of weaned piglets was better when we fed dietary FF, and the ADFI of weaned piglets in the test group (FF) tended to increase compared with the control group (GF). In addition, the ADG of weaned piglets in the FF group was significantly higher, and the F: G, diarrhea incidence and mortality were significantly lower compared with the control group.

**Table 4:** Effects of dietary FF on growth performance in weaned piglets (*n* = 120).

**Item**	**GF**	**FF**
ADFI/g	861.52 ± 64.28	893.94 ± 32.51
ADG/g	502.06 ± 31.81^b^	569.48 ± 36.47^a^
F: G	1.72^a^	1.56^b^
Diarrhea incidence/%	25.81 ± 0.05^a^	7.32 ± 0.23^b^
Mortality/%	10.78 ± 0.42^a^	3.61 ± 0.18^b^

a, b Within a row, the letter means without a common superscript difference (*p* < 0.05); No letter means the difference is not significant (*p* > 0.05).

### 3.4. Immunity properties

**Figure 5:**
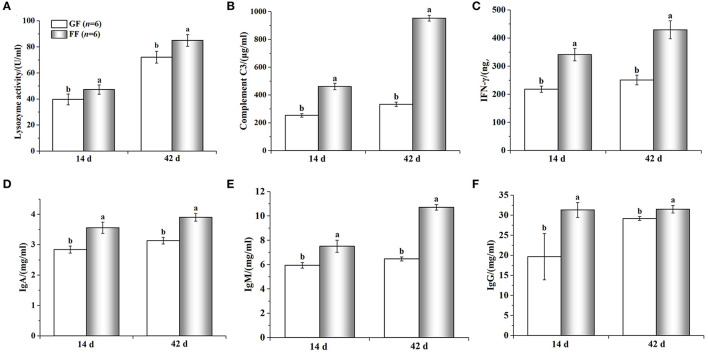
Effects of dietary FF or GF on immunologic factors [**(A)**: Lysozyme activity; **(B)**: Complement C3; **(C)**: IFN-γ; **(D)**: IgA; **(E)**: IgM; **(F)**: IgG] in the serum of piglets.

The experimental results (Figure 5) showed that the six immune indexes in the serum of piglets in the FF group at day 14 and 42 of age were higher than those in the control group. Among them, serum lysozyme activity in the FF group was higher than that in the GF group; both serum complement C3 and IFN-γ levels in the FF group at day 14 and 42 were significantly higher than those in the group GF (*p* < 0.05); levels of serum IgA and IgM in the FF group at day 14 were higher than those in the GF group, and the content of IgA and IgM in the serum of piglets in the FF group were significantly higher than those in the GF group at day 42 (*p* < 0.05); the content of serum IgG in the FF group at day 14 were significantly higher than those in the GF group.

### 3.5. Fecal microbiota

**Figure 6:**
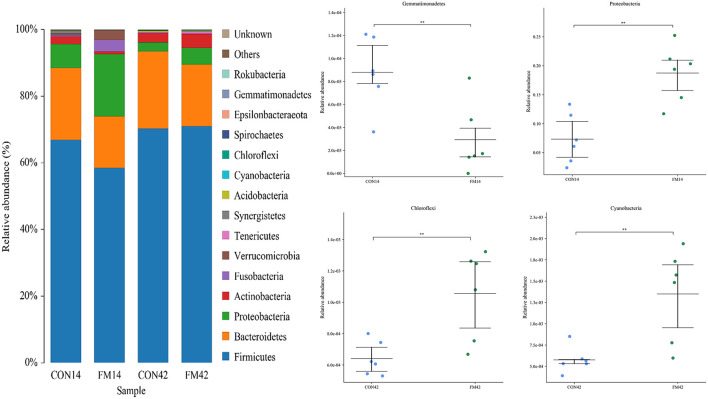
Fecal microbiota richness and diversity at phylum level (*n* = 6). The left side of the figure shows the composition of the bacteria, and the right side shows the bacteria with extremely significant differences at phylum level.

At the phylum level, *Firmicutes, Bacteroidetes, Proteobacteria* and *Actinobacteria* were the dominant bacteria, which accounted for 90% of bacteria in the GF and FF group (Figure 6). Compared with the GF group, at day 14, the abundance of *Firmicutes, Bacteroidetes* and *Gemmatimonadetes* (*p* < 0.01) decreased and that of *Proteobacteria* (*p* < 0.01) increased in the FF group. At day 42, the abundance of *Bacteroidetes* decreased and that of *Proteobacteria* (*p* < 0.05), *Chloroflexi* (*p* < 0.01) and *Cyanobacteria* (*p* < 0.01) increased in the FF group, while that of *Firmicutes* was stable.

**Figure 7:**
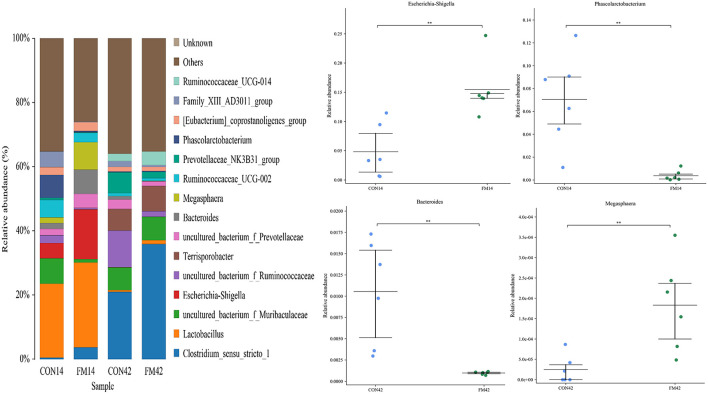
Fecal microbiota richness and diversity at genus level (*n* = 6). The left side of the figure shows the composition of the bacteria, and the right side shows the bacteria with extremely significant differences at genus level.

At the genus level, the dominant genera of piglet fecal microorganisms in each group were *Clostridium_sensu_stricto_1, Lactobacillus* (*p* < 0.05), *Escherichia-Shigella* (*p* < 0.01), *Terrisporobacter, Bacteroides* (*p* < 0.05), etc (Figure 7). Compared with the GF group, the abundance of *Clostridium_sensu_stricto_1, Lactobacillus* and *Escherichia-Shigella* in the feces of piglets increased and that of *Phascolarctobacterium* (*p* < 0.01) decreased at day 14 in the FF group. The abundance of *Clostridium_sensu_stricto_1* and *Lactobacillus* in the feces of piglets increased and that of *Bacteroides* (*p* < 0.01), *Megasphaera* (*p* < 0.01) decreased at day 42 in the FF group.

**Figure 8:**
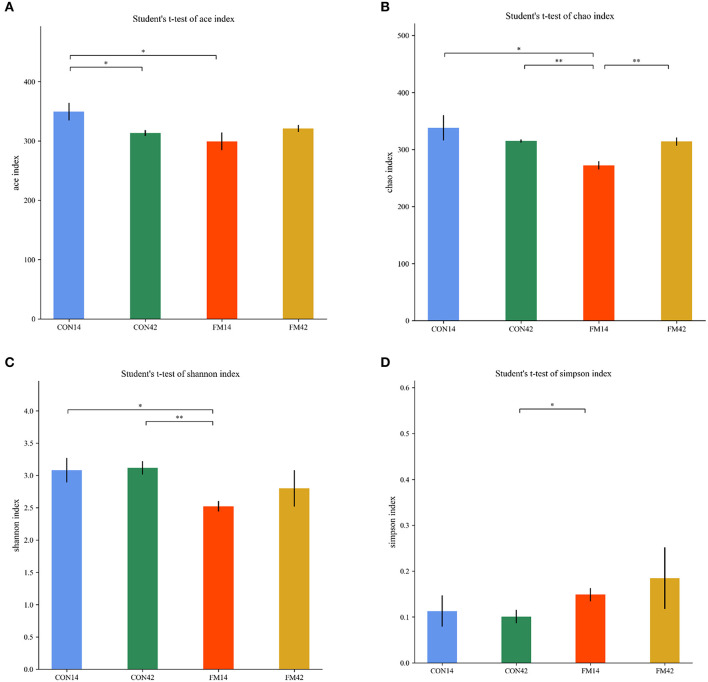
Comparison of fecal microbiota structure by α-diversity [**(A)**: ace; **(B)**: chao; **(C)**: shannon; **(D)**: simpson].

The α-diversity of the fecal microbiota of piglets including Chao, Ace, Simpson and Shannon indexes were presented in Figure 8. The FF diet induced lower Chao, Ace and Shannon indexes than the GF at day 14 (*p* < 0.05). It indicated a decrease in the number and diversity of fecal microbial species in piglets that were FF by diet-induced. However, lower difference was shown in the indexes at day 42 (*p* < 0.05).

**Figure 9:**
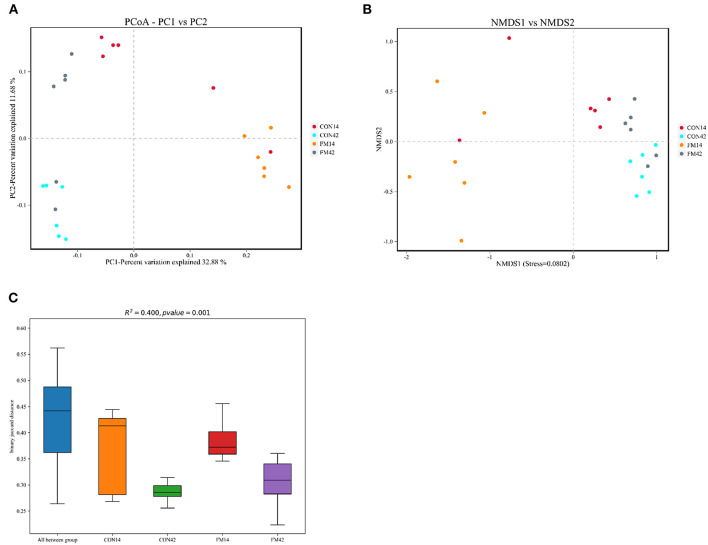
Comparison of fecal microbiota structure by β-diversity [**(A)**: PCoA; **(B)**: NMDS; **(C)**: box plot].

The β-diversity of bacterial community between GF and FF was presented, which all showed a tendency for different clustering of microbial communities in feces (Figure 9). In general, FF had a large effect on the fecal microbial diversity of 14-day-old piglets, but a relatively small effect on the fecal microbial diversity of 42-day-old piglets.

## 4. Discussion

SBM is the main protein resource for animal production; however, some anti-nutritional factors such as soybean antigenic proteins and trypsin inhibitor reduce their nutritional value and inhibit animal production (6). Soybean antigenic proteins have been shown to cause antigenic protein hypersensitivity, which is attributed to gut injury, inflammation, and diarrhea (7). Fermented feed is a novel of green feed to replace antibiotics, studied by researchers and fed to animals in many countries widely. During the fermentation process, fermented feed can effectively degrade anti-nutritional factors and toxins, promoting digestion and absorption of nutrients, improving the immunity of animals and preventing animal diseases, reducing breeding pollution, etc. (8–10). Our study used corn, bran and SBM as the main fermented substrates by inoculating with an effective combination of probiotics.

Certainly, the nutritional quality of the fermented feed depends on the type and combination of probiotics used for inoculation. In present study, we used the three probiotics mixed fermentation, and the optimal combination of them was then investigated which used soybean antigenic proteins as an indicator (5, 11, 12). After fermentation, feed has lower pH value, trypsin inhibitor, glycinin and β-conglycinin, and higher content of crude protein and acid-soluble protein. This is of great importance for the healthy growth of weaned piglets. The lower pH value was mostly due to the lactic and acetic acid produced by *L. casei* and *S*. *cerevisiae*, which stimulates chewing rate and helps eliminate total hydrogen ions in the gastric and intestine, and inhibits the reproduction of harmful microorganisms and minimizes the loss of nutrients (13–15). The lower content of anti-nutritional factors could be attributed to *B. licheniformis*, which can secrete lots of enzymes, such as aminopeptidases, serine endopeptidases, metalloproteinases, and neutral proteases, which are able to decompose proteins (16). And it can colonize the intestinal tract as a dominant flora, maintaining the intestinal microecological balance, which has a better effect on improving the intestinal health and growth performance of weaned piglets. Additionally, in the process of mixed culture fermentation, there is a complementary and mutually constraining relationship between the probiotics. Pyruvic acid, carbon dioxide and other substances were produced by the metabolism of *B. licheniformis* and *S. cerevisiae* not only provide an anaerobic environment for the growth of *L. casei*, but also provide nutrients to promote the activity of *L. casei*, which in turn metabolize and produce organic acid and other substances. The increase of crude protein and acid-soluble protein could be that the plant-based proteins in FF are metabolically utilized by probiotics to convert them into bacteriophage proteins, improving palatability, facilitating the digestion or absorption of piglets, thus ensuring the utilization and quality of FF.

Many previous studies have also confirmed that animals fed with fermented feeds showed better growth performance than animals fed with conventional feeds, such as geese (17), broilers (18), shrimps (19), sea cucumber (20), and so on. In the study of the apparent metabolism of weaned piglets, we could find an upward trend in ADFI, ADG, and a lower F: G. The reason might be that the fermentation process degrades the complex macromolecules such as starch, cellulose and protein in the feed into small molecules such as monosaccharides, disaccharides, oligosaccharides and amino acids which are easily digested and absorbed by piglets, and produces microbial proteins and microbial metabolites. The reduction in diarrhea rates and mortality might be the beneficial microbes existing in FF to inhibit the pathogenic microbial proliferation (21–23).

A study reported that the dietary supplementation of FF increased serum IgM and IgG levels compared with GF (23). The results of the present study were consistent with this finding. Pătrascanu et al. reported that IgM, IgG and lysozyme activity showed a significantly increase when the probiotics were supplemented in pigs (24). Similarly, our results were also like this, indicating the probiotics had immune stimulatory action. The increase of INF-γ could be attributed to *lactobacillus*. Some studies found that *lactobacillus* can enhance macrophage activity and local antibody levels, then induce the production of interferon and anti-inflammation cytokines (25–27), or improve immunity by modulating the functions of intestinal epitheliocytes (PIE) (27–29). In the presence of FF, the levels of all immune molecules in pig serum were increased. This might be due to the presence of probiotics and organic acids in the fermented feed, which inhibited pathogens or mycotoxins, enhanced intestinal development and reduced postweaning diarrhea (30, 31). These could also be the reason for increased immunity and low diarrhea incidence and mortality in weaned piglets.

For the fecal microbiota of weaned piglets, at the phylum level, *Firmicutes* and *Bacteroidetes* were the dominant bacteria. Compared with GF, the relative abundance of *Bacteroidetes* was lower both at day 14 and 42 in the presence of FF. It might be due to the fact that the *L. casei* in the fermented feed lowed the pH of the intestine, thus inhibiting the colonization and multiplication of Gram-negative bacteria such as *Bacteroidetes* (32). The relative abundance of the phylum *Chloroflexi* was dramatically promoted by the FF compared to that in the GF at day 42. This could be explained by the increase of organic acids in the intestine, which promoted the development of the genus *Chloroflexus* among them. At the genus level, fermented feed could selectively reduce the abundance of *Bacteroides*, and our result was consistent with previous studies (33). In addition, we found that FF significantly increased the abundance of *Megasphaera*, which is beneficial for intestinal functions and partly prevented diarrhea on weaned piglets (34). The α-diversity index reflects the richness and diversity of the microbial community in the sample (35). Our study found that FF could significantly decrease the Shannon and Chao index of feces in weaned piglets, especially at day 14 (Figures 8B, C). It might be attributed to the fact that the *L. casei* in the FF changed the structure of the intestinal flora of the piglets, which in turn improved digestion, absorption and organism health (36, 37). In addition, as shown in Figures 8C, D, we found that the diversity of the fecal microbiota in weaned piglets fed FF decreased at both day 14 and 42. This result might be owing to the fermentation of the feed by probiotics (*L. casei*), which led to an increase in the content of *Lactobacillus* indirectly and a decrease in the proportion of their dominant bacteria (Figure 7). The β-diversity results did reveal a significant difference between FF and GF, especially on day 14. Predictably, the intestinal flora stabilizes as the piglets grow, and the effect of FF decreases on weaned piglets gradually.

## 5. Conclusions

Taken together, mixed probiotics fermentation with *B. licheniformis, S. cerevisiae* and *L. casei* effectively reduced anti-nutritional factors in mixed feed and increased the content of crude protein, acid-soluble protein and organic acid. Moreover, FF had advantages over GF in promoting the performance of weaned piglets, which was mainly reflected by the increased ADFI, ADG and decreased F: G, diarrhea, mortality. Compared with GF, FF improved immunity *via* increased serum levels of immunological molecules. Furthermore, FF modulated fecal microbiota composition and metabolites including a lower abundance of *Bacteroidetes* and a higher abundance of *Lactobacillus*. In conclusion, the appropriate addition of feed fermented by *B. licheniformis, S. cerevisiae* and *L. casei* could enrich beneficial bacteria and further regulate the balance of intestinal flora, promoting the immunity and health status of growing piglets, having good application prospects.

## Data availability statement

The datasets presented in this study can be found in online repositories. The name of the repository and accession number can be found below: NCBI; PRJNA921733.

## Ethics statement

The animal study was reviewed and approved by Ethics Committee of Experimental Animal Management, School of Medicine, Huaqiao University.

## Author contributions

DJ, LL, and JJ conceived and designed the experiments. JX and LD contributed to the animal feeding and results analysis. DJ drafted the manuscript. LL, YS, LZ, CH, and MY were responsible for the modification of manuscript. All authors contributed to the article and approved the submitted version.
