# Corrigendum: Three-stage fermentation of the feed and the application on weaned piglets

**DOI:** 10.3389/fvets.2023.1230874

**Published:** 2023-06-13

**Authors:** Dahai Jiang, Manqi Yang, Jun Xu, Liping Deng, Cong Hu, Liangliang Zhang, Yunzhang Sun, Jianchun Jiang, Liming Lu

**Affiliations:** ^1^Academy of Advanced Carbon Conversion Technology, Huaqiao University, Xiamen, China; ^2^College of Chemical Engineering, Huaqiao University, Xiamen, China; ^3^Zhangzhou DaBeiNong Agriculture and Husbandry Science & Technology Co., Ltd., Zhangzhou, China; ^4^Jiangxi DaBeiNong Technology Co., Ltd., Nanchang, China; ^5^Beijing DaBeiNong Technology Group Co., Ltd., Beijing, China; ^6^The Key Laboratory of Healthy Mariculture for the East China Sea, Ministry of Agriculture, Fisheries College, Jimei University, Xiamen, China; ^7^Institute of Chemical Industry of Forest Products, CAF, Nanjing, China

**Keywords:** probiotics, nutritional value, weaned piglets, growth performance, fermented feed

In the published article, there was an error. The terms “glycine” and “β-glycine” were used when it should have been “glycinin” and “β-conglycinin”.

A correction has been made to the **Abstract**, paragraph 1. The sentence previously stated:

“Trypsin inhibitor, glycine and β-glycine were reduced by 79.86, 77.18, and 69.29%, respectively.”

The corrected sentence appears below:

“Trypsin inhibitor, glycinin and β-conglycinin were reduced by 79.86, 77.18, and 69.29%, respectively.”

The authors apologize for this error and state that this does not change the scientific conclusions of the article in any way. The original article has been updated.

